# Characterization of the Reduced IgE Binding Capacity in Boiled and Autoclaved Soybeans through Proteomic Approaches

**DOI:** 10.3390/foods11030479

**Published:** 2022-02-07

**Authors:** Xiaowen Pi, Yuxue Sun, Xiaomin Deng, Dawei Xin, Jianjun Cheng, Mingruo Guo

**Affiliations:** 1 College of Food Science, Northeast Agricultural University, Harbin 150030, China; 13361617637@163.com (X.P.); sunffyy@163.com (Y.S.); xiaomindeng98@126.com (X.D.); xdawei@163.com (D.X.); 2 Key Laboratory of Soybean Biology of Chinese Education Ministry, Harbin 150030, China; 3 Department of Nutrition and Food Science, College of Agriculture and Life Sciences, University of Vermont, Burlington, VT 05405, USA

**Keywords:** autoclaving, boiling, allergen, peptide, IgE binding capacity

## Abstract

The study investigated the changes in IgE binding capacity, protein profiles and peptide compositions after soybeans were boiled and autoclaved. The results of ELISA showed that the IgE binding capacity of soybean was reduced by 69.3% and 88.9% after boiling and autoclaving, respectively. Above 43 and 10 kDa proteins disappeared in boiled and autoclaved soybeans from SDS-PAGE, respectively. A Venn diagram and heat map showed that there was no change in allergen types and a reduction in allergen contents in the boiled and autoclaved soybeans. The changes in peptide compositions were also observed in the boiled and autoclaved soybeans through Venn diagram, PCA and heat map. LC/MS-MS and peptide mapping analysis demonstrated that boiling and autoclaving masked many epitopes in Gly m 4 and Gly m 5, such as ALVTDADNVIPK, SVENVEGNGGPGTIKK and KITFLEDGETK of Gly m 4 and VEKEECEEGEIPRPRPRPQHPER of Gly m 5, resulting in a reduction of IgE binding capacity in the extracted proteins. By contrast, the exposure of many epitopes in Gly m 6 was observed in boiled and autoclaved soybeans, which might be mainly responsible for the existing IgE binding capacity in the treated soybean proteins. Interestingly, the IgE binding capacity of soybeans showed a positive correlation with the total contents and number of peptides in Gly m 4–Gly m 6.

## 1. Introduction

Soybean (*Glycine max*) is a good source of nutritional protein for animal and human consumption due to its high protein content (dry weight basis, 34.1–56.8%) [1], but it is one of eight major food allergens, affecting 1.4% of the referred population [2,3]. Generally, soybean allergy can cause allergic reactions, including rhinitis, urticarial, vomiting, shock and death, etc. [2]. Moreover, the LOAEL (the lowest amount of the allergenic food inducing allergic symptoms) is 5.3 mg of soybean proteins [4]. Currently, many soybean allergens have been identified, including Gly m 1 to Gly m 8, P28, P34 and kunitz trypsin [2,5,6]. All soybean allergens account for approximately 65–80% of total soybean proteins and 30% of defatted soybean meal [7]. Gly m 4 (17 kDa), Gly m 5 (42–53 kDa, 57–76 kDa, 57–83 kDa), Gly m 6 (56, 54, 54, 64, and 59 kDa), P28 (28 kDa), P34 (30 kDa) are the major soybean allergens [2]. P34, P28 and Gly m 5 are derived from the 7S globulin, whereas Gly m 6 is from 11S globulin and Gly m 4 is a pathogenesis-related protein [2].

Boiling and autoclaving are common thermal technologies used to inactivate microorganism and anti-nutritional factors of food products with low cost and easy use [2,8,9,10]. Many studies have demonstrated that the allergenicity of soybeans and peanuts were successfully reduced by boiling and autoclaving, respectively [6,11,12,13,14]. Our previous study has also shown that the allergenicity of soybeans was decreased by boiling and autoclaving, resulting from the degradation of proteins and the changes in conformation [10]. However, the changes of peptide compositions and the modification of epitopes in boiled and autoclaved was not investigated.

LC/MS-MS is a common and effective way of investigating the mechanism of processing technologies to reduce the allergenicity of allergens [15,16,17,18]. Liu et al. used an Orbitrap LC-MS/MS to evaluate *D*-(+)-ribose glycated *β*-lactoglobulin with ultrasound pretreatment and found that ultrasound pretreatment promoted the reduction in allergenicity by improving the glycation extent of some glycation sites [17]. Similar results were shown by Yang et al., who found that 22, 21, 18, 16 and 13 glycated sites had been determined in ovalbumin (OVA)-*D*-allose, OVA-*D*-galactose, OVA-*L*-idose, OVA-*D*-mannose and OVA-*D*-glucose by HPLC-Orbitrap-MS analysis, respectively, which was responsible for the reduction in allergenicity in glycated ovalbumin [16]. Proteomics approaches based on the Orbitrap LC/MS-MS are usually used for the differences in protein composition and function [19,20]. Sun et al. found that colostrum milk showed more functions associated with protein processing in the endoplasmic reticulum, whereas mature milk had more oxidative phosphorylation functions using a data-dependent acquisition technique based on the Orbitrap LC/MS-MS [19]. Similar results were shown in saanen goat milk from different provinces in China using a data-independent acquisition technique based on the Orbitrap LC-MS/MS, which showed that the proteins of goat milk from Guangdong and Shaanxi were most similar in composition [20]. In addition, QTRAP LC-MS/MS was successfully used for simultaneous quantification of allergens in surimi products with the limitation of 0.054 μg/g for soybean, 0.024 μg/g for milk and 0.032 μg/g for egg [21]. Yoshimitsu et al. further used an QTRAP LC-MS/MS to find that the content of Gly m 4 was 309–421 µg/g in soybean grains and 2.7–67 µg/g in soybean-processed foods [22]. Therefore, LC/MS-MS has been widely used in the analysis and detection of allergens. At present, there are few reports that investigate the characterization of the reduced allergenicity in boiled and autoclaved soybean using LC/MS-MS-based proteomics methods. This study analyzed the effects of thermal-sterilized technologies (boiling and autoclaving) on allergenicity, protein profiles and peptide compositions of soybeans by proteomics approaches based on the Orbitrap LC/MS-MS to establish the relationship between allergenicity and protein or peptide compositions, providing the foundation for producing hypoallergenic soybean products.

## 2. Materials and Methods

### 2.1. Experimental Materials

Soybeans (*Glycine max* (Linn.) Merr) were from Northeast Agricultural University. Five patients’ sera with the IgE levels of 5.484, 12.088, 38, 47 and 72.333, kU/I in soybean allergens were from PlasmaLab (Everett, WA, USA). The information of patients was shown in Table S1. Goat anti-human IgE HRP conjugates and 3,3′, 5,5′-tetramethylbenzidine solution were both purchased from Chongqing Manuik Technology Co., Ltd. (Chongqing, China). Pierce C18 tips were purchased from Thermo Fisher Scientific (Waltham, MA, USA). All other chemical reagents were obtained from Sigma-Aldrich Co. (St. Louis, MO, USA) and the corresponding solutions were prepared using Milli-Q-treated water (Millipore, Bedford, MA, USA).

### 2.2. Pre-Treatment of Boiled and Autoclaved Soybeans

In order to obtain boiled and autoclaved soybeans, 100 g of raw soybeans were soaked in 2 L of distilled water for 12 h and then drained for 2 min, while other soybeans were boiled and autoclaved in water at 100 °C and in a tabletop autoclave at 121 °C for 20 min.

### 2.3. Preparation of Soybean Proteins

Soybean proteins was prepared using our previously reported methods [10]. In brief, the soybeans were crushed by grinder and then degreased with n-hexane three times. After defatted soybean powder was air-dried overnight, the soybean proteins were extracted with distilled water of pH 8.5 three times. The mixture of three extracts produced soybean proteins for the next analysis. The content of proteins was evaluated through the bicinchoninic acid (BCA) [23].

### 2.4. Determination of IgE Binding Capacity by Indirect ELISA

The IgE binding capacity of the protein extracts was assessed by indirect ELISA in accordance with [24]. Five patients’ sera were pooled together to be used as an IgE+ serum. Each sample (2 μg/mL protein, 100 μL) was coated in microtiter plate wells overnight. After the wells were washed by PBS with 0.05% Tween-20 (PBST) three times, 100 μL skim milk was added in the wells to block for 60 min at 37 °C. After washing, 100 μL IgE+ serum was added in the wells to incubate for 1 h at 37 °C. The IgE+ serum was removed and the wells were washed by PBS with 0.05% Tween-20 three times. An amount of 100 μL of diluted goat anti-human IgE HRP conjugates was added in the wells and incubated at 37 °C for 1 h. After washing, an amount of 100 μL of 3,3′, 5,5′-tetramethylbenzidine solution was added in the wells to incubate for 13 min at 37 °C. Finally, an amount of 100 μL H_2_SO_4_ solution (0.2 M) was added to stop the reaction, and then the absorbance was measured at 450 nm. The IgE binding capacity (%) = OD_450_ (other samples)/OD_450_ (Raw soybeans).

### 2.5. SDS-PAGE

SDS-PAGE was performed with 12% separating gel (running voltage, 120 V) and 5% stacking gel (running voltage, 60 V) at denaturing conditions [10]. An amount of 40 μL of protein extracts (0.65 mg/mL) was diluted with 10 μL loading buffer containing *β*-mercaptoethanol (Beyotime, Shanghai, China), and then boiled for 3 min. The loading volume of the resulting solution was 5 μL (2.5 μg proteins). Then, the gels were stained with Coomassie Brilliant Blue R-250G. Finally, the gels were destained with deionized water and scanned with image scanner III (ODYSSEY cLx, LI-COR, Lincoln, NE, USA).

### 2.6. LC/MS-MS

The procedures of LC/MS-MS were performed from [19]. Soybean proteins (150 μg) were dissolved with 100 μL of 50 mM NH_4_HCO_3_ solution, reduced by 2 μL of 500 mM dithiothreitol and then alkylated by 14 μL of 500 mM iodoacetamide. After the resulting samples were filtered by 10-kDa cutoff filter and washed twice with pH 8.5 buffer solution (8 M urea in 100 mM Tris/HCl), the retentate sample was dissolved with 100 μL of 50 mM NH_4_HCO_3_ solution and then digested with trypsin at 37 °C for overnight. An 8 μL of C_2_HF_3_O_2_ solution (10%, *v*/*v*) was added to end the digested reaction. The resulting peptides were desalted by C18 tips, then vacuum dried. The dried samples were dissolved with 20 μL of 0.1% formic acid solution. Four microliters of the resuspended sample were injected into a C18 pre-Column (5 μm, 100 μm × 2 cm). Then, the sample was separated on a C18 analytical column (3 μm, 75 μm × 100 nm) with a linear gradient of mobile phase (0.1% formic acid in 80% acetonitrile). The flow rate was 300 nL/min. After that, the resulting sample was analyzed by Q ExactivePlus-Orbitrap MS (Thermo Fisher Scientific) at a range of 350–2000 *m*/*z*, 1.8 kV spray voltage and 300 °C heater temperature. MS/MS was conducted at a range of 200–2000 *m*/*z*, 27 eV collision energy and 60 s dynamic exclusion.

### 2.7. Statistical Analysis

The data are expressed as the mean ± standard deviation. The analysis was performed using Origin-Pro 8.0 (OriginLab Corp., Northampton, MA, USA) and SPSS (International Business Machines Corporation, Windows 2003, Armonk, NY, USA). LC-MS/MS data were bottom-up analyzed using Proteome Discoverer 24 Software (Thermo Fisher Scientific) under the soybean database (https://www.uniprot.org/, accessed on 13 December 2020). The abundance of protein and peptide was assessed by the label-free quantification (LFQ) method with untreated samples as the standard [25]. Briefly, precursor ion areas were calculated using a Precursor Ion Area Detector and normalized by the Total Peptide Amount mode in Proteome Discoverer 2.2. A Venn diagram, peptide map, PCA map and heat map were drawn by some online tools (e.g., https://bioinfogp.cnb.csic.es/tools/venny/index.html (accessed on 1 September 2021). http://bioware.ucd.ie/peptigram (accessed on 1 September 2021) and https://biit.cs.ut.ee/clustvis (accessed on 1 September 2021).

## 3. Results and Discussion

### 3.1. The IgE Binding Capacity of Boiled and Autoclaved Soybeans

**Figure 1:**
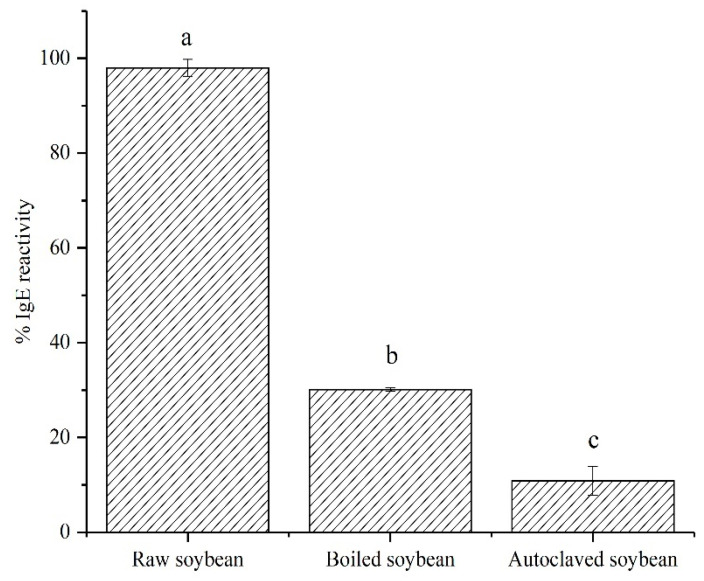
Changes in IgE binding capacity of extracted proteins from raw, boiled and autoclaved soybeans. Means with different letters (**a**–**c**) indicate significantly different (*p* < 0.05).

The IgE binding capacity of soybeans was assessed by indirect EILSA using mixed allergic patients’ serum. There were significant (*p* < 0.05) differences in IgE binding capacity among raw, boiled and autoclaved soybean proteins (Figure 1). Compared to extracted proteins from raw soybeans, the IgE binding capacity of boiled and autoclaved soybean proteins was decreased by 69.3% and 88.9%, respectively. The IgE binding capacity of autoclaved soybean proteins was lower than boiled soybean proteins, probably resulting from the high temperature and pressure for autoclaving, compared to the simple boiling procedure. Similar studies have also demonstrated that the allergenicity of heated egg white and silkworm pupa protein extract were reduced with the increase of heating time and temperature, respectively [24,26]. High temperature caused high protein aggregation and denaturation [27,28] to result in the destruction of epitopes and the reduction of allergen contents in extracted soybean proteins, which was responsible for the reduction of IgE binding capacity in boiled and autoclaved soybean proteins (Figure 1). The reduction of IgE binding capacity was also probably due to the masking and destruction of epitopes induced by the changes of conformation and the degradation of proteins [23,29,30]. Kim et al. found that the degradation of porcine serum albumin was responsible for the decrease of allergenicity in boiled and autoclaved porcine serum albumin [30]. Zhang et al. also demonstrated that the allergenicity of Ara h 2 was reduced after the peanuts had boiled for 15 min due to the reduction of core epitope binding capacity induced by the changes in the protein structure [29]. In addition, the allergenicity of the autoclaved soybeans was not completely reduced, probably resulting from the existence of epitopes [31]. Besides, autoclaving may induce glycation to create neo-epitopes that are able to activate an IgE response [32]. In conclusion, boiling and autoclaving could induce various reactions (e.g., aggregation denaturation, degradation of proteins and glycation) to result in the changes in protein profiles and peptide compositions responsible for the changes in IgE binding capacity of soybean proteins, which need to be investigated.

### 3.2. Changes in Protein Profiles of Extracted Protein

**Figure 2:**
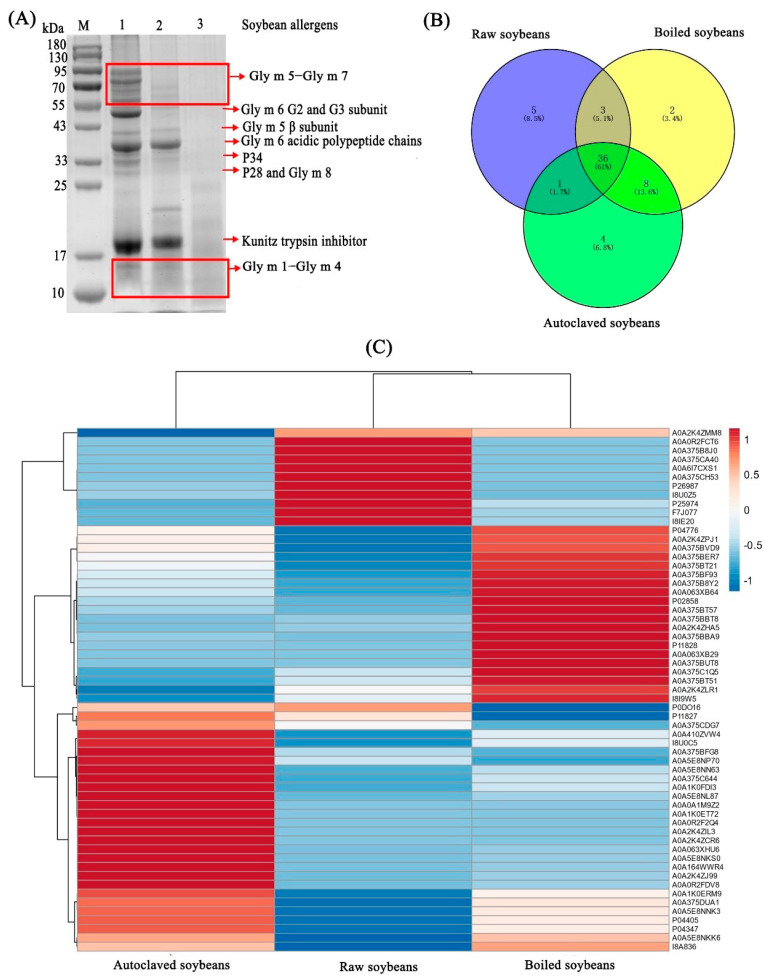
Changes in protein profiles of extracted proteins from raw, boiled and autoclaved soybeans. (**A**) SDS-PAGE, M, standard proteins; lands M, 1, 2 and 3 represented extracted proteins from raw, boiled and autoclaved soybeans, respectively. (**B**) Venn diagrams. (**C**) heat map and cluster analysis, the deeper the red band, the higher the relative abundance of the protein, and the deeper the blue, the lower the relative abundance.

As shown in Figure 2A, boiled soybean showed less intensity of stained bands (molecular weight, MW > 43 kDa) but an increase of low molecular weight bands (molecular weight <43 kDa) (land 2), compared to protein bands in raw soybeans. This is consistent with Cabanillas et al., who found that boiled peanuts showed less intensity of stained bands (50 kDa < MW< 81 kDa) but an increase of intensity in stained bands (19 kDa < MW < 50 kDa) [13]. By contrast, the protein bands ≥10 kDa showed less intensity after soybeans were autoclaved (Figure 2A, land 3). Similar studies have also demonstrated that there were fewer protein bands in autoclaved peanuts than in boiled peanuts, and the band intensity of autoclaved peanut proteins was reduced with the increase of temperature and time [14]. Boiling and autoclaving could induce protein aggregation and denaturation [27,28], which was responsible for the disappearance of protein bands due to the reduction of extracted proteins. In addition, boiling and autoclaving caused the conformational changes in soybean proteins [10] to result in the separation of polypeptide chains, which might be responsible for the disappearance, shallow of high MW protein bands and the increase of low MW protein bands. It was reported that protein aggregation and denaturation could induce the decrease in protein solubility and the changes in protein structure to result in the loss of allergens and the destruction/masking of epitopes [10], which was responsible for the reduction of IgE binding capacity in boiled and autoclaved soybean proteins (Figure 1). What is more, based on Table S2, it was observed in a reduction of allergens such as Gly m 5-Gly m 7 and kunitz trypsin inhibitor among boiled and autoclaved soybean proteins. Therefore, the lower concentration of soybean allergens was observed in autoclaved soybean proteins, which might be responsible for a lower allergenicity than boiled soybean proteins.

The differences in protein profiles can be further analyzed by Venn diagram and heat map. The results of the Venn diagram demonstrated that there were 45, 49 and 49 proteins grouped from raw, boiled and autoclaved soybean proteins, respectively (Figure 2B). Boiling and autoclaving could result in the exposure of some amino acid residue by structural changes [10], probably resulting in an increase of protein amounts in boiled and autoclaved soybeans. There were five (UniProtKB, A0A375B8J0, A0A0R2FCT6, A0A375CH53, A0A6I7CXS1, A0A375CA40), two (A0A063XB29, A0A375BUT8) and four (A0A0R2F2Q4, A0A1K0ET72, A0A2K4ZIL3, A0A2K4ZCR6) unique proteins in raw, boiled and autoclaved soybean proteins, respectively (Figure 2B). Compared to raw soybean proteins, there were eight (A0A375BT21, A0A375BVD9, A0A1K0ERM9, A0A410ZVW4, A0A5E8NKS0, A0A063XHU6, A0A375CDG7 and A0A0A1M9Z2) unique proteins in boiled and autoclaved soybean proteins. However, these unique proteins were not allergens according to Table S3 and the World Health Organization and International Union of Immunological Societies (WHO/IUIS) Allergen Nomenclature Subcommittee. In addition, there were 36 common proteins in all samples, in which 10 (P25974, P11828, P11827, P04776, P04405, P0DO16, F7J077, P26987, P04347 and P02858) common proteins were identified as SAM22, beta-conglycinin and glycinin. It was reported that SAM22, beta-conglycinin and glycinin were considered as Gly m 4, Gly m 5 and Gly m 6, respectively, accounting for approximately 70% of the total soybean proteins [2]. Therefore, boiling and autoclaving were difficult to completely remove soybean allergens by protein aggregation and denaturation. The differences of protein profiles were furtherly analyzed by a heat map (Figure 2C). It was showed that the protein in raw and boiled soybeans was similar, which was consistent with SDS-PAGE (Figure 2A). In addition, it was observed that there were significant differences in protein distribution and content, resulting for protein aggregation, denaturation and structural changes in boiled and autoclaved soybeans.

### 3.3. Changes in Identified Soybean Allergens

**Figure 3:**
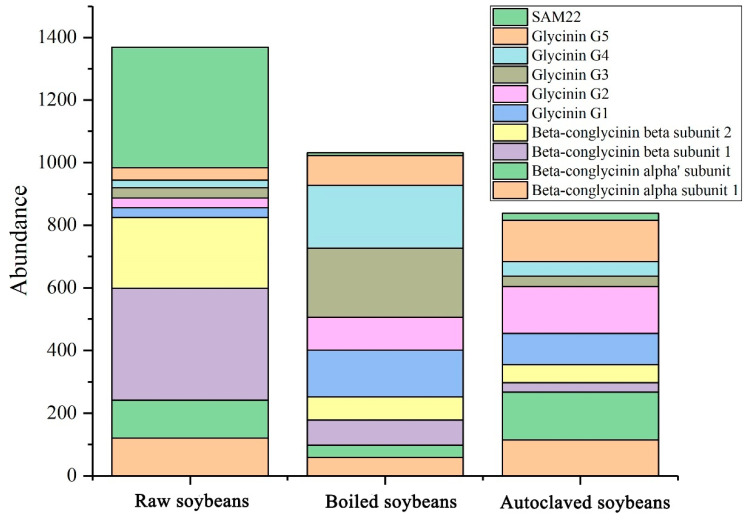
Changes in contents of identified allergens (Gly m 4-Gly m 6) in extracted proteins from raw, boiled and autoclaved soybeans.

Figure 3 demonstrated that soybean allergens in raw, boiled and autoclaved soybean proteins were identified by LC/MS-MS, including Gly m 4 (SAM22), Gly m 5 (Beta-conglycinin) and Gly m 6 (glycinin). It was observed that there was the lowest content of total identified allergens in autoclaved soybean, followed by boiled and raw soybeans, which was consistent with the results of SDS-PAGE (Figure 2A). The loss of soybean allergens was due to the decrease in protein solubility [13,33]. In addition, the reduced abundance of SAM22 and beta-conglycinin in boiled and autoclaved soybeans were probably due to their masking induced by structural changes, resulting in the difficult degradation by trypsin. By contrast, the increased abundance of glycinin suggested the exposure of glycinin. The exposure of glycinin could contribute to the reaction with the enzyme, making the protein more easily degraded into peptides that were detected by LC/MS-MS, which was responsible for the increased abundance of glycinin. It was reported that Gly m 4, Gly m 5 and Gly m 6 were major soybean allergens and recognized by IgE in 96%, 86% and 86% of patients allergic to soybean, respectively [2,34], which were responsible for severe reactions for patients allergic to soybeans [35]. Thus, the reduction and masking of allergens, especially Gly m 4 and Gly m 5, were responsible for the decrease of allergenicity in boiled and autoclaved soybean proteins. However, the allergenicity was not completely reduced, probably resulting from the exposure of Gly m 6.

### 3.4. Changes in Peptide Profiles in Identified Soybean Allergens

**Figure 4:**
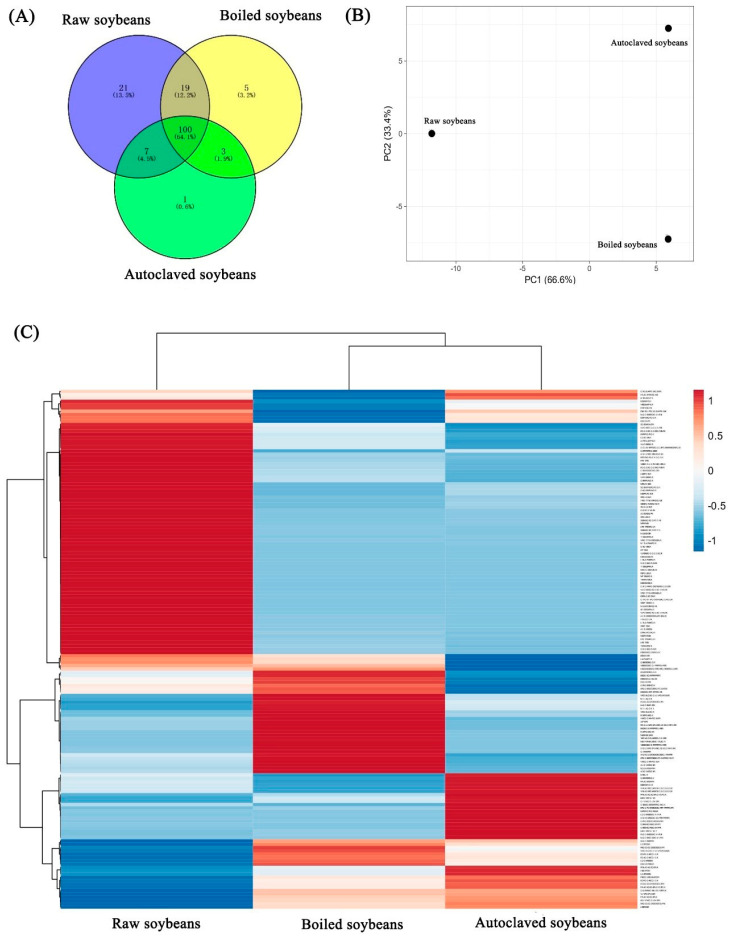
Differences of peptide compositions in identified soybean allergens (Gly m 4−Gly m 6). (**A**) Venn diagrams. (**B**) PCA analysis. (**C**) heat map and cluster analysis, the deeper the red band, the higher the relative abundance of the protein, and the deeper the blue, the lower the relative abundance.

Figure 4A demonstrated the changes in peptide numbers among Gly m 4, Gly m 5 and Gly m 6. The peptide number was decreased from 147 to 127 and 111 after the raw soybeans were boiled and autoclaved, respectively; their changes were consistent with the changes of allergenicity (Figure 1). Similar results were shown in unique peptide numbers, which showed 21, 5, and 1 unique peptide in raw, boiled and autoclaved soybean proteins, respectively. Thus, the decrease of allergenicity in boiled and autoclaved soybean proteins might be due to the decrease in number of total or unique peptides. The differences of peptides were furtherly analyzed by PCA and heat map (Figure 4B,C). Figure 4B showed that there was the lowest distance between boiled and autoclaved in PC1 (66.6%), suggesting the similar peptides in boiled and autoclaved, which might be responsible for the similar allergenicity between boiled and autoclaved soybeans. Similar results were found in the heat map (Figure 4C). Thus, compared to raw soybeans, the content and compositions of peptides in Gly m 4-Gly m 6 were significantly changed after raw soybeans were boiled and autoclaved.

**Figure 5:**
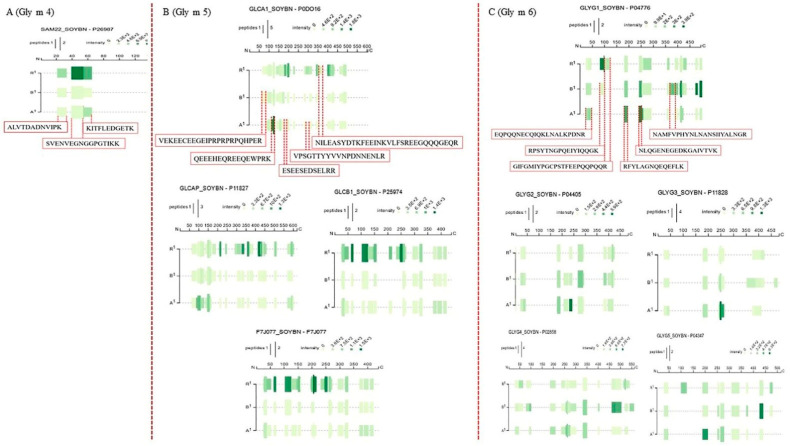
Changes in peptide map of Gly m 4-Gly m 6 in extracted proteins from raw (R1), boiled (B1) and autoclaved soybeans (A1). (**A**) peptide map of Gly m 4 (UniProtKB, P26987). (**B**) peptide map of Gly m 5, including beta-conglycinin alpha subunit 1 (UniProtKB, P0DO16), beta-conglycinin alpha’ subunit (P11827), beta-conglycinin beta subunit 1 (P25974) and beta-conglycinin beta subunit 2 (F7J077). (**C**) peptide map of Gly m 6, including Glycinin G1(P04776), G2 (P04405), G3 (P11828), G4 (P02858) and G5 (P04347). The relative abundance, amino acid coverage and overlap ratio was shown by the color depth, the width and length of each band, respectively.

A peptide map was used to investigate the changes of peptide in Gly m 4, Gly m 5 and Gly m 6, respectively. Figure 5A demonstrated that there was the reduced relative abundance of Gly m 4 (SAM22) in boiled and autoclaved soybean proteins, in which boiled soybean proteins showed lower abundance in ALVTDADNVIPK (AA22-33), SVENVEGNGGPGTIKK (AA40-55), KITFLEDGETK (AA55-65) than autoclaved soybean proteins. The reduced abundance of peptides suggested the masking of peptides, resulting from structural changes. Many epitopes in Gly m 4 are on surfaces, accounting for 80% of total IgE binding capacity of Gly m 4 [2,36]. Our previous study has shown the changes in protein structure in boiled and autoclaved soybeans proteins [10]. Therefore, boiling and autoclaving induced structural changes to mask ALVTDADNVIPK, SVENVEGNGGPGTIKK and KITFLEDGETK in Gly m 4 might cause the decrease of allergenicity. For Gly m 5 (Beta-conglycinin alpha subunit 1, Beta-conglycinin alpha’ subunit, Beta-conglycinin beta subunit 1, Beta-conglycinin beta subunit 2), there was significant differences in peptide profiles among raw, boiled and autoclaved soybeans (Figure 5B). A reduced abundance or absence of peptides were observed in the boiled and autoclaved soybean proteins, compared to raw soybean proteins, suggesting a great possibility of masking peptides. Gly m 5 contains three subunits: the *α* subunit, *α*’ subunit and *β* subunit, in which the allergenicity of α subunit (Gly m 5.0101) is strongest [2]. 68.1% of epitopes in Gly m 5 are located in the surface of the *α* subunit [37]. As shown in Gly m 5.0101 (UniProtKB, P0DO16), there was some absence of peptides in the boiled and autoclaved soybeans, involving ESEESEDSELRR (AA178-189), VPSGTTYYVVNPDNNENLR (AA287-305), NILEASYDTKFEEINKVLFSREEGQQQGEQR (AA340-370), suggesting their masking. Thus, boiling and autoclaving reduced the allergenicity by masking these peptides containing epitopes. In addition, there was an increased abundance and absence for VEKEECEEGEIPRPRPRPQHPER (AA63-85) in boiled and autoclaved soybeans, respectively. Epitope (AA55-80) in α subunit of Gly m 5 was the main epitope [38]. Thus, the decrease of allergenicity in autoclaved soybean proteins was higher than in boiled soybean proteins, probably resulting from the masking and exposure of VEKEECEEGEIPRPRPRPQHPER (AA63-85) in autoclaved and boiled soybean proteins, respectively. By contrast, an increased abundance for QEEEHEQREEQEWPRK (AA113-128) and QEEEHEQREEQEWPRK (AA113-128) were observed in autoclaved soybeans, which was not consistent with the changes in allergenicity. Thus, these peptides (AA113-128, AA113-128) might not be epitopes or low allergic epitopes. For Gly m 6 (glycinin G1, G2, G3, G4, G5), there were many increased abundances of peptides and new peptides in boiled and autoclaved soybeans (Figure 5C), suggesting the exposure of many peptides. Thus, many epitopes in Gly m 6 were exposed, which might be responsible for the existence of allergenicity in the boiled and autoclaved soybeans. Epitopes in allergens play important roles in inducing allergic reaction [2,29]. Allergic peptides in identified soybean allergens were observed and concluded in Table S4 based on the previous study [2] and online site (http://www.uwm.edu.pl/biochemia/index.php/en/biopep) (accessed on 1 September 2021). Table S4 demonstrated that the total content of epitopes in Gly m 4 and Gly m 5 was decreased after soybeans were boiled and autoclaved, suggesting the masking of many epitopes in Gly m 4 and Gly m 5. Therefore, boiling and autoclaving reduce the soybean allergenicity by masking many epitopes in Gly m 4 and Gly m 5. The allergenicity of soybeans was not completely reduced due to the exposure of many epitopes in Gly m 6 and the existence of partial epitopes in Gly m 4 and Gly m 6.

## 4. Conclusions

The IgE binding capacity of soybeans was decreased after boiling and autoclaving, which was accompanied by the changes in protein profiles and peptide compositions due to protein denaturation, aggregation and structural changes. The loss of soybean allergens and the masking of many epitopes in Gly m 4 and Gly m 5 were responsible for the decrease of IgE binding capacity in boiled and autoclaved soybean proteins. Therefore, boiling and autoclaving might be effective ways of producing hypoallergenic soybean products, although this needs to be proved by more clinical trials. Nevertheless, LC/MS-MS is an effective auxiliary method for ELISA to assess soybean allergies, providing the sequence and quantitative information of the epitope or peptide.

## Data Availability

Data is contained within the article.

## Supplementary Materials

The following are available online at https://www.mdpi.com/article/10.3390/foods11030479/s1, Table S1: Table S1 Information of patients allergic to soybeans; Table S2: The molecular weight of soybean allergens (Amnuaycheewa & de Mejia, 2010; Pi, Sun, Fu, et al., 2021); Table S3: The information of proteins; Table S4: Changes in epitopes in Gly m 4 and Gly m 5 among extracted proteins from raw, boiled and autoclaved soybeans.
