# Supplementary material for: Characterization of the Reduced IgE Binding Capacity in Boiled and Autoclaved Soybeans through Proteomic Approaches
Source: Foods. 2022 Feb 7;11(3):479. doi: 10.3390/foods11030479 (PMC8834289; doi:10.3390/foods11030479)
Supplement: Supplementary file 1 [file foods-11-00479-s001.zip › foods-1480036-supplementary/Table S1-S4.pdf]

Table S1. Information of patients allergic to soybeans

Sample	Gender	Ethnicity	Birth Date	Soybean allergen IgE levels (kU/I)
1	Female	Caucasian	04/01/1979	5.484
2	Female	American	09/09/1992	72.333
3	Female	American	09/09/1992	38
4	Male	Asian	10/14/1993	12.088
5	Female	American	09/09/1992	47.7

Table S2. The molecular weight of soybean allergens (Amnuaycheewa & de Mejia, 2010; Pi, Sun, Fu, et al., 2021)

Soybean allergens	Molecular weight (kDa)
Gly m 1	8.3
Gly m 2	8
Gly m 3	14
Gly m 4	17
Gly m 5	α subunit: 57-76<br>α' subunit: 57-83<br>β subunit: 42-53
Gly m 6	G1 subunit: 56<br>G2 subunit: 54<br>G3 subunit: 54<br>G4 subunit: 64<br>G5 subunit: 59
Gly m 7	76.2
Gly m 8	28
Gly m Bd 28K (P28)	28
Gly m Bd 30K (P34)	30
Kunitz trypsin inhibitor	20

Table S3. The information of proteins

Accession	Description	Coverage [%]	MW [kDa]
P0DO16	Beta-conglycinin alpha subunit 1	54	70.3
P11827	Beta-conglycinin alpha' subunit	51	72.2
P04776	Glycinin G1	51	55.7
P25974	Beta-conglycinin beta subunit 1	50	50.4
F7J077	Beta-conglycinin beta subunit 2	50	50.4
P02858	Glycinin G4	49	63.8
P04347	Glycinin G5	35	57.9
P04405	Glycinin G2	33	54.4
P11828	Glycinin G3	29	54.2

P26987	Stress-induced protein SAM22	24	16.8
A0A0R2FDV8	DNA-binding protein HU	13	10
A0A0A1M9Z2	30S ribosomal protein S5	12	17.9
A0A5E8NL87	Elongation factor Tu	11	43.4
A0A375C1Q5	Elongation factor Tu	10	43.1
A0A063XHU6	50S ribosomal protein L19	9	13.4
A0A5E8NN63	6-phosphogluconate dehydrogenase (Decarboxylating)	8	32.7
A0A2K4ZCR6	50S ribosomal protein L15	8	15.6
A0A5E8NNK3	Pyruvate kinase	7	50.4
A0A375BT21	30S ribosomal protein S13	7	13.6
A0A375BF93	Iron-sulfur cluster assembly scaffold protein IscU	7	14.1
A0A063XB64	50S ribosomal protein L5	7	20.1
A0A063XB29	Elongation factor Tu	7	43.6
A0A375C644	2-dehydro-3-deoxy-phosphogluconate aldolase	6	21.6
I8IE20	Calmodulin	5	17
A0A6I7CXS1	Ribonuclease YeeF	5	21.6
A0A5E8NKS0	ATP-dependent Clp protease ATP-binding subunit	5	76.9
A0A410ZVW4	Chaperone protein DnaK	4	65.9
A0A375DUA1	Response regulator in two-component regulatory system with CopS, regulation of copper resistance	4	25.6
A0A375BT57	50S ribosomal protein L2	4	30.1
A0A375BBT8	ATP synthase subunit beta	4	50.8
A0A2K4ZIL3	30S ribosomal protein S2	4	28.2
I8U0Z5	Molecular chaperones HSP70/HSC70, HSP70 superfamily	3	69.7
A0A375CH53	Elongation factor G	3	77.8
A0A375CDG7	Putative typeIsecretion system outer membrane efflux transmembrane protein	3	53.4
A0A375CA40	Flagellin	3	44.9
A0A375BFG8	Ketol-acid reductoisomerase (NADP(+))	3	36.6
A0A164WWR4	Trigger factor	3	47.5
I8U0C5	Elongation factor 1-alpha	2	50
I8I9W5	ATP synthase subunit alpha	2	59.9
A0A5E8NP70	Glucose-6-phosphate 1-dehydrogenase	2	55.1
A0A5E8NKK6	Glyceraldehyde-3-phosphate dehydrogenase	2	35.7
A0A375BVD9	60 kDa chaperonin	2	57.4

A0A375BUT8	Putative ACYL-COA DEHYDROGENASE OXIDOREDUCTASE	2	65.3
A0A375BER7	Succinate-semialdehyde dehydrogenase I, NADP-dependent	2	51.5
A0A375BBA9	Succinate--CoA ligase [ADP-forming] subunit beta	2	41.2
A0A375B8Y2	ATP synthase subunit alpha	2	55.4
A0A375B8J0	Adenosylhomocysteinase	2	51.9
A0A2K4ZPJ1	Neopullulanase	2	71.6
A0A2K4ZLR1	Formate acetyltransferase	2	76.1
A0A1K0FDI3	Phosphoketolase	2	92.2
A0A1K0ET72	Trigger factor	2	46.3
A0A1K0ERM9	Phosphoglycerate kinase	2	43.1
A0A0R2FCT6	GTP-binding protein	2	68.4
A0A0R2F2Q4	ATP synthase subunit beta	2	50.3
I8A836	Enoyl reductase domain of FAS1	1	198.4
A0A375BT51	DNA-directed RNA polymerase subunit beta'	1	155.8
A0A2K4ZMM8	Chaperone protein HtpG	1	106.7
A0A2K4ZJ99	ATP-dependent Clp protease ATP-binding subunit ClpC	1	90.8
A0A2K4ZHA5	DNA-directed RNA polymerase subunit beta'	1	140.1

Table S4. Changes in epitopes in Gly m 4 and Gly m 5 among extracted proteins from raw, boiled and autoclaved soybeans

Amino acid sequence	Abundance			Allergens
	Raw soybean	Boiled soybean	Autoclaved soybean	
ALVTDADNVIPK	367.7	10.5	19.7	Gly m 4
SVENVEGNGGPGTIK	407.4	8.6	5	
SVENVEGNGGPGTIKK	514.3	12.9	23	
VEKEECEEGEIPRPRPRPQHPER	0	29.4	0	α subunit of Gly m 5
EECEEGEIPRPRPRPQHPER	1.4	22.1	0	
EPQQPGEKEEDEDEQPRPIPFPRPQPR	62.6	76.2	259.2	
EEDEDEQPRPIPFPRPQPR	56.2	88.4	6.1	
EEQEWPRKEEK	32.5	88.1	65.8	
EEQEWPR	88.9	59.5	80.4	
EEQEWPRK	29.8	41.8	17.5	
GSEEEDEDEDEEQDER	311.2	56.4	14.7	
QFPFPRPPHQK	78.7	30.3	259.1	
QFPFPRPPHQKEER	137.7	5.2	32	
KQEEDEDEEQQR	68.6	129.7	36.1	
QEEDEDEEQQR	135.4	108.4	0	

ESEESEDSELR	616	0	0	
ESEESEDSELRR	575.7	0	0	
NKNPFLFGSNR	477.1	8.2	56.4	
NPFLFGSNRFETLFK	566.4	0	0	
NPFLFGSNR	296.2	3.3	29.1	
LQSGDALR	165.6	58.2	25.9	
VPSGTTYYVVNPDNNENLR	572.9	0	0	
VLFSREEGQQQGEQR	309.9	0	0	
KTISSEDKPFNLR	783.9	0	0	
TISSEDKPFNLR	548.8	1.4	4.3	
SRDPIYSNK	491.5	12.7	2.8	
DPIYSNK	437.8	0	0	
FFEITPEKNPQLR	473.8	19.3	6	
FFEITPEK	283.2	37.9	16.1	
EQQQEQQQEEQPLEVRK	183.6	52.7	0	
EQQQEQQQEEQPLEVR	195.3	33.7	11.7	
ESYFVDAQPK	308.9	0	153.1	
VEEEEECEEGQIPRPRPQHPER	54.7	53	6.2	α’ subunit of Gly m 5
QQHGEKEEDEGEQPRPFPFPRPR	8	2.3	845.1	
EEDEGEQPRPFPFPRPR	29.8	64.6	9.3	
EHPRPHQPHQKEEEK	14.2	8.6	370.3	
EEEKHEWQHK	108.3	81.6	187.7	
EEEKHEWQHKQEK	54.2	102.5	0	
ESEEEEEDQDEDEEQDK	207.6	9.9	0	
VLFGREEGQQQGEER	87.9	28.1	0	
KTISSEDKPFNLR	783.9	0	0	
TISSEDKPFNLR	548.8	1.4	4.3	
LFEITPEKNPQLR	543.5	12.4	4.7	
LFEITPEK	334.1	34.9	22.8	
QQQEEQPLEVR	272.7	26.9	14.4	
QQQEEQPLEVRK	361.6	0	0	
LAIPVNKPGR	118.6	102.6	27	*β*-subunit 1 of Gly m 5
QQEGVIVELSK	843.7	0	20.5	
TISSEDEPFNLR	610.9	0	0	
SRNPIYSNNFGK	883.6	0	0	
NPIYSNNFGK	495.4	0	0	
FFEITPEKNPQLR	473.8	19.3	6	
FFEITPEK	283.2	37.9	16.1	
QKQEEEPLEVQR	295.9	50.3	75.6	
QEEEPLEVQR	342.7	70.6	18.3	
AILTLVNNDDRDSYNLHPGDAQR	834.1	2.4	0	*β*-subunit 2 of Gly m 5
AILTLVNNDDR	467	17.1	7.4	
LAIPVNKPGR	118.6	102.6	27	
VLLGEEEEQR	240.5	74.7	17.5	
VLLGEEEEQRQQEGVIVELSK	663.2	0	0	
QQEGVIVELSK	843.7	0	20.5	
TISSEDEPFNLR	610.9	0	0	
SRNPIYSNNFGK	883.6	0	0	

NPIYSNNFGK	495.4	0	0	
FFEITPEKNPQLR	473.8	19.3	6	
FFEITPEK	283.2	37.9	16.1	
QKQEEEPLEVQR	295.9	50.3	75.6	
QEEEPLEVQR	342.7	70.6	18.3	
Total	23888.7	2076.7	2940.7	
